# Improved image quality in subtraction based non-contrast MRA using automated soft tissue motion correction with BRACE

**DOI:** 10.1186/1532-429X-11-S1-P84

**Published:** 2009-01-28

**Authors:** Zhaoyang Fan, Peter Weale, Xiaoming Bi, James Carr, Saurabh Shah, John Sheehan, Debiao Li, Renate Jerecic

**Affiliations:** 1 Northwestern University, Chicago, IL USA; 2 Siemens Medical Solutions, Chicago, IL USA

**Keywords:** Image Quality, Motion Correction, Anterior tibiaL, Posterior Tibial, Automate Motion

## Introduction

Recently several new approaches for non-contrast MRA have been proposed. Many of them are based on subtraction of two 3D data sets, which are acquired either at two different points of the cardiac cycle, eg NATIVE SPACE or use different preparation mechnisms to alter the signal intensity in the arteries and veins, eg by flow sensitizing dephasing (FSD)gradients. As the acquisition of each dataset takes in the order of 2–3 minutes, motion can occur in between the two acquisitons and can impair therefore the resulting image quality. A common problem which is also known from contrast enhanced MRA, where the mask is subtracted form the contrast data set [1].

## Purpose

To evaluate in volunteers and patients whether an automated soft tissue motion correction software can improve the image quality of subtraction based non-contrast MRA data.

## Methods

Non contrast peripheral MRA data sets of the lower legs of 3 healthy volunteers and 3 patients where qualitatively scored before and after motion correction by an experienced radiologist. The commecially available software *syngo* BRACE (Siemens Healthcare, Germany) was used [1] to perform the automated motion correction of the two data sets.

Qualitative image quality was assed using a scale from 1 to 4 (1 being poor, 2 fair, 3 good, 4 excellent vessel conspicuity) The conspicuity was scored for the popliteal, anterior tibial, posterior tibial and the peroneal branch as well as for the side branches. A score was given for each branch before and after motion correction.

## Results

**Figure 1:**
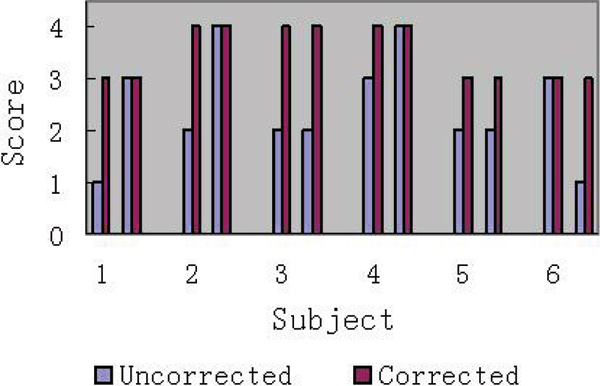
**Scores of the anterior tibial artery on uncorrected images vs. corrected images in 6 subjects**. Scores were improved in all legs with appreciable motion, but unchanged in all still.

**Figure 2:**
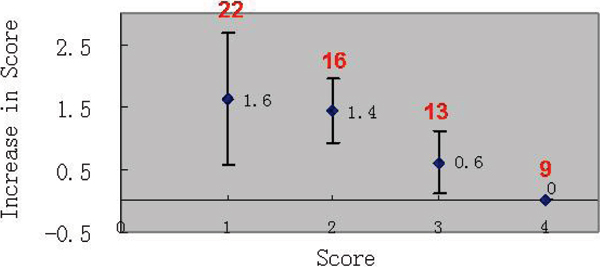
**Absolute improvement in image scores for groups with initial score of 1, 2, 3 and 4, respectively**. All legs (60 segments) were pooled together. (Red figures: the total number of each group). Vessel depiction of segments with initial score of 1 or 2 (primarily due to motion) were substantially improved after motion correction.

**Figure 3:**
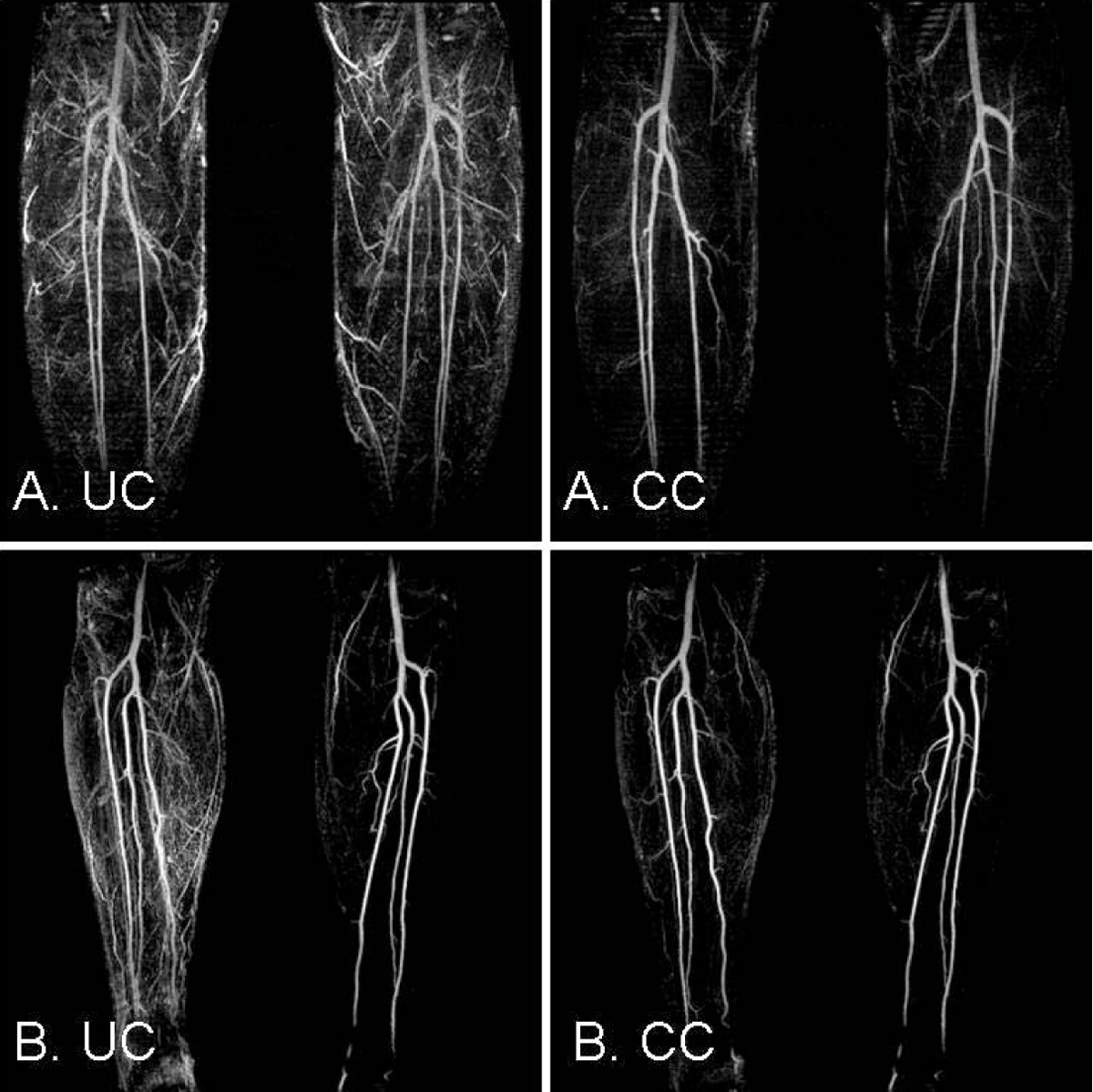
**Image quality and artery depiction was improved after correction when motion was present in both legs (A) or only one leg in which case correction didn't affect the originally still leg (B)**. UC uncorrected; CC: corrected.

The results are shown in Figure 1 on the example of the anterior tibiaL branch assesment. The asessment of the other branches was comparable. Figure 2 shows the overall improvement based on the initial score before motion corredtion was applied. Figure 3 shows two typical case examples before and after BRACE was applied.

## Conclusion

The motion correction software BRACE was able to improve the image quality in subtraction based non contrast MRA techniques when motion occued. Many cases showed that motion often occurs in only one leg. In those cases BRACE did not deteriorate the image quality of the leg without motion while at the same time improving the result in the leg affected by motion. No case was observed where the software did decrease the image quality compared to the uncorrected image. BRACE therefore provides a promising tool in the area of non contrast MRA.
